# Impact of negative air pressure in ICU rooms on the risk of pulmonary aspergillosis in COVID-19 patients

**DOI:** 10.1186/s13054-020-03221-w

**Published:** 2020-09-01

**Authors:** Philippe Ichai, Faouzi Saliba, Patricia Baune, Asma Daoud, Audrey Coilly, Didier Samuel

**Affiliations:** 1 Liver Intensive Care Unit, Centre Hépato-Biliaire, AP-HP, Hôpital Paul-Brousse, Université Paris-Saclay, Inserm U1193, 12 Avenue Paul Vaillant Couturier, F-94804 Villejuif, France; 2 Infection Control Team, AP-HP Hôpital Paul-Brousse, F-94800 Villejuif, France

From the start of the COVID-19 epidemic, one recommendation regarding the intensive care management of COVID-19 patients concerned the infrastructure in intensive care units (ICU) and particularly the air pressure system in ICU rooms [1]. Under normal circumstances and mainly in ICUs hosting immunocompromised patients, ICU rooms are equipped with positive air room pressure in order to protect patients against infections from the surrounding environment and particularly those due to *Aspergillus fumigatus* (AF). This link between positive pressure rooms and a reduction in *Aspergillus* infection rates has been demonstrated during several studies [2]. However, these studies included numerous other associated preventive measures. The relationship between the fungal levels in the air of neutral pressure rooms and those in positive pressure rooms has not been established [3].

During the current COVID-19 pandemic, on the contrary, the recommendations have been to place intensive care rooms under negative or even normal pressure so as to protect the staff and patients healthcare. Two recent studies reported a high incidence (26.3–33%) of pulmonary aspergillosis in COVID-19 infected patients [4, 5]. This high risk of pulmonary aspergillosis was also seen in patients with severe influenza (19%). Thevissen et al. reported that the rate of influenza-associated pulmonary aspergillosis (IAPA) varied according to country and that variation in IAPA prevalence might be related to underdiagnosis due to lower use of galactomannan testing on broncho-alveolar lavage or serum in some areas [6].

In line with these recommendations, the 15 rooms of our ICU were placed under negative pressure to receive COVID-19 patients. During this change, all filters in the air conditioning unit were replaced. Two months earlier, routine air sampling had not revealed the presence of any fungal agents and our annual incidence of aspergillosis was lower than 2%. Between 23 March and 4 May 2020, 26 COVID-19 patients were admitted to our ICU (Table 1). Six of the 26 (23.1%) developed probable or proven pulmonary aspergillosis, while two were colonized by AF.

**Table 1:** Principal characteristics of patients with probable or proven pulmonary aspergillosis

Mean age (SD): 64 ± 9 years Gender (*n* males): 18 M ARDS due to COVID-19, *n* = 21 Delay between admission and the diagnosis of aspergillosis: 6.5 ± 4.2 days Antifungal therapy: 6/6 - Isavuconazole, *n* = 5 - Voriconazole, *n* = 1 Alive: 4/6 patients (67%)	

Air cultures from the rooms occupied by the first four infected patients revealed the presence of AF. No colonies of AF were found on the surfaces in rooms or in the air of stepdown rooms (same building, same geographical orientation, two floors up) that were sampled at the same time and were being operated under unventilated rooms. During the crisis, no building projects were being carried out anywhere near the ICU. Checks on accessible sections of the room ventilation circuits did not identify any reservoir of contamination.

After surface disinfection, the negative air pressure in the rooms was raised on two successive occasions, ultimately reaching a pressure of 1.2 ± 1.5 Pa. From that time on, the levels of AF in room air fell in spectacular fashion (0–2 CFU/m^3^) (Fig. 1).

**Fig. 1:**
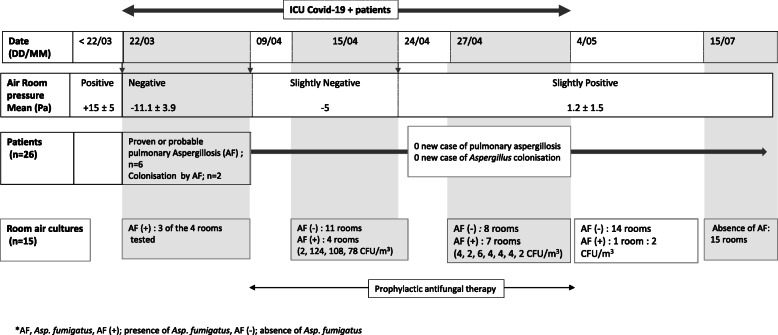
Chronology of *Aspergillus* infections among COVID-19 patients in the ICU and environmental mycological results. After lowering to a negative pressure in the 15 ICU rooms, probable or proven pulmonary aspergillosis developed in six patients and *Aspergillus* colonization in two patients. Analysis of the air in the three of four rooms tested, before any corrective measures, were positive for *Aspergillus fumigatus.* Despite decreasing the negative air pressure to − 5 Pa, high levels of *Asp. fumigatus* remained positive in the room air. When the air pressure in the rooms was brought to around 0 Pa, the number of *Aspergillus* colonies markedly diminished and then became undetectable. Since then, no patient developed *Asp. fumigatus* infection. AF, *Asp. fumigatus*; AF (+), presence of *Asp*. *fumigatus*; AF (−), absence of *Asp*. *fumigatus*

All infected patients received antifungal therapy and 4 out of 6 are alive. Prophylactic antifungal therapy was administered to all other patients. Since then, no further cases of aspergillosis have been recorded.

Our results demonstrate that implementing negative pressure in ICU rooms could be the source of air contamination by *Aspergillus* and thus increase the risk of opportunistic infections. A switch to neutral or slightly positive pressure in the rooms, combined with standard environmental cleaning protocols and prophylactic antifungal treatments, enabled the eradication of aspergillus from the air in these rooms. One hypothesis regarding contamination was a spread of dust from the plenum spaces in false ceilings which “might” have been moved during successive adjustments to high/low/neutral pressure and could have infiltrated via unsealed parts of the ceiling.

Close mycological screening of COVID-19 infected patients (biomarkers and mycological diagnosis) and regular controls of air quality are highly recommended.

## Data Availability

If necessary, data could be transmitted to the Editor.
